# Closed‐Loop Automated Insulin Delivery in Patients With Type 2 Diabetes: A Meta‐Analysis of Randomised Controlled Trials

**DOI:** 10.1002/edm2.70178

**Published:** 2026-03-13

**Authors:** Dua Ali, Abdur Rafay Bilal, S. M. Washaqul Arfin, Maryam Sajid, Shaheer Qureshi, Raheel Ahmed, Hasibullah Aminpoor, Saad Ahmed Waqas

**Affiliations:** ^1^ Department of Medicine Dow University of Health Sciences Karachi Pakistan; ^2^ Ziauddin Medical University Karachi Pakistan; ^3^ Academic Clinical Lecturer Newcastle University Newcastle upon Tyne UK; ^4^ Freeman Hospital Newcastle upon Tyne UK; ^5^ Royal Brompton Hospital London UK; ^6^ Faculty of Medicine Kabul University of Medical Sciences “Abu Ali Ibn Sina” Kabul Afghanistan

## Abstract

**Aims:**

To evaluate the efficacy and safety of closed‐loop automated insulin delivery (AID) systems with the conventional insulin delivery arm in adults with type 2 diabetes mellitus (T2DM).

**Methods:**

A systematic review and meta‐analysis of 10 randomised controlled trials (*n* = 786; AID: 436, control: 350) was conducted per PRISMA guidelines. Data from studies were pooled using a random‐effects model to derive weighted mean differences (WMDs) and 95% confidence intervals (CIs).

**Results:**

AID significantly increased time in normoglycemia (WMD: +22.14%; 95% CI: 17.05, 27.23; 319 min/day; P < 0.00001) and reduced hyperglycemia (WMD: −21.04%; 95% CI: −6.23, −15.84; −303 min/day; P < 0.00001), mean glucose (WMD: −1.54 mmol/L; 95% CI: −2.37, −0.70; P = 0.0003), and HbA1c (WMD: −0.80%; 95% CI: −1.48, −0.12; P < 0.00001). Glucose SD improved significantly (WMD: –0.46; 95% CI: −0.74, −0.18; P = 0.002). No significant differences were found in hypoglycemia (WMD: −0.11%; 95% CI: −0.32, 0.09; −1.6 min/day; P = 0.28), total daily insulin (WMD: +4.80 IU; 95% CI: −1.87, 11.47; P = 0.16), or CV (WMD: −0.52%; 95% CI: −2.08, 1.04; P = 0.52). Total adverse events were higher with AID (RR: 1.61; 95% CI: 1.20, 2.17; P = 0.002), but serious adverse events (RR: 1.61; 95% CI: 0.83, 3.13; P = 0.16) and severe hypoglycemia (OR: 2.13; 95% CI: 0.22,20.83; P = 0.52) were not significantly different.

**Conclusions:**

Closed‐loop AID significantly improves glycemic control in T2DM without increasing serious adverse events.

## Graphical abstract

Comparative efficacy and safety of closed‐loop automated insulin delivery (AID) systems with the conventional insulin delivery arm in adults with type 2 diabetes mellitus (T2DM).

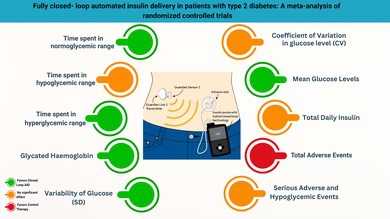

## 1 Introduction

Globally, type 2 diabetes mellitus (T2DM) affects approximately 462 million individuals, representing 6.28% of the world's population, that is projected to rise to 853 million by 2050 [1, 2]. T2DM is a complex metabolic disorder characterised by insulin resistance and progressive β‐cell dysfunction, often requiring insulin therapy for optimal glycemic control [3]. Despite numerous pharmacological options, maintaining glucose within target ranges remains challenging due to risks of hypoglycemia, regimen complexity, and patient nonadherence [4]. Traditional insulin regimens demand frequent monitoring and manual dose adjustments, contributing to suboptimal outcomes in many patients with T2DM [5].

Automated insulin delivery (AID) systems, also known as closed‐loop systems, represent a significant technological advancement in diabetes management. These systems integrate continuous glucose monitoring (CGM) with algorithm‐driven insulin pumps, allowing dynamic insulin adjustments based on real‐time glucose levels [6]. While AID systems have demonstrated substantial benefits in individuals with type 1 diabetes, their role in the management of T2DM has only recently begun to be explored [7]. Unlike type 1 diabetes, where insulin deficiency is the primary issue, T2DM patients present with complex pathophysiology, making personalised AID algorithms potentially even more critical [8, 9].

A previous meta‐analysis evaluating AID in T2DM patients was constrained by limited trial data, reducing its statistical power and clinical applicability [10]. However, the field has rapidly evolved, with several randomised controlled trials (RCTs) recently published that offer a more robust evidence base. This updated systematic review and meta‐analysis incorporates these new data to comprehensively assess the efficacy, safety, and patient‐centered outcomes of AID systems in T2DM. In particular, this review also seeks to explore whether AID can reduce glycemic variability, improve time‐in‐range metrics, and lower the burden of hypoglycemia, emerging therapeutic targets that go beyond simple HbA1c reduction. By bridging current evidence gaps, this study aims to inform therapeutic decision‐making and support the broader integration of AID technologies in the management of T2DM.

## 2 Methodology

This systematic review and meta‐analysis were conducted according to the guidelines set by Cochrane and the Preferred Reporting Items for Systemic Reviews and Meta‐Analyses (PRISMA) [11, 12]. Permission from an ethical review board was not required, as the data used were publicly available. The study protocol has been registered on PROSPERO with the ID number provided (PROSPERO ID: CRD420251091165).

### 2.1 Data Sources and Search Screening

A comprehensive digital search was conducted in the PubMed and Cochrane CENTRAL and Scopus databases from their inception until April 10, 2025, without applying any filters or language restrictions. The search strategy is outlined in Table S1. We used the snowballing approach from relevant systematic reviews to ensure no critical publication was overlooked. All retrieved articles were exported to Rayyan.ai for the identification and removal of duplicates [13]. The selection process occurred in two stages: an initial screening based on titles and abstracts, followed by a full‐text review using predefined eligibility criteria. Both stages were conducted independently by two reviewers (DA and SMWA). Any disagreements were resolved by consulting a third reviewer (ARB).

### 2.2 Eligibility Criteria and Study Selection

We included studies that met the following inclusion criteria: (1) randomised controlled trials (RCTs), (2) comparisons of AID with conventional subcutaneous insulin, (3) patients with T2DM, and (4) studies that reported at least one of the pre‐specified outcomes: Our primary outcome was the proportion of time spent in the target glucose concentration (100–180 mg/dL or 5.6–10 mmol/L); however, for studies that did not report this range, 3.9–10 or 3.9–8 mmol/L were used. Key secondary outcomes included the proportion of time spent in hyperglycemia (sensor glucose measurements > 10 mmol/L) and that spent in hypoglycemia (sensor glucose measurements < 5.6 mmol/L). Other outcomes included total daily insulin, mean glucose level, Glycated Haemoglobin (HbA1c), glucose variability (SD), Coefficient of Variation (CV) in Glucose Level and safety measures.

### 2.3 Data Extraction and Quality Assessment

Trial characteristics, baseline demographics, outcomes, and safety data were extracted by the authors (DA and SMWA) onto a pre‐designed Excel spreadsheet. The quality assessment of the included trials was conducted using the Cochrane Risk of Bias tool by two reviewers (DA and ARB) [14]. No specific concerns were noted regarding the imprecision or indirectness of the results, as the studies included were well‐designed and appropriately powered.

### 2.4 Statistical Analysis

For continuous outcomes, mean change from baseline (with standard deviation [SD]) to endpoint, between the treatment and placebo groups, to calculate the effect sizes. For dichotomous outcomes, we employed risk ratios (RRs) and odds ratios (OR) using the Mantel–Haenszel method. All analyses were performed using Review Manager (Version 5.4; Copenhagen: The Nordic Cochrane Centre, The Cochrane Collaboration, 2020). To maintain consistency across studies, we computed the mean difference (MD) using Review Manager for those that reported only pre‐ and post‐treatment values separately for each group. In cases where studies presented mean differences with either 95% confidence intervals (CIs) or standard errors (SEs), these were converted to mean differences with SDs using the same software.

We pooled effect sizes using a random‐effects model to derive mean differences (MDs) with corresponding 95% confidence intervals (CIs). Forest plots were created to visually represent the analyses. To assess the heterogeneity among the trials, we used Higgins *I* ^*2*^. We categorised *I* ^*2*^ values as follows: 25% to 50% was considered mild, 50% to 75% was moderate, and values over 75% were classified as severe [15]. A P‐value of < 0.05 was considered statistically significant. The Chi‐square test was performed to assess differences between the subgroups. Subgroup analyses explored whether factors such as Daytime versus Overnight and follow‐up durations influenced the effect size on the glycemic ranges. Additionally, we conducted a sensitivity analysis excluding specific studies to identify any outlier studies in the forest plot. Review Manager (Version 5.4, Copenhagen: The Nordic Cochrane Centre, The Cochrane Collaboration, 2020) was used for all statistical analyses. Publication bias was assessed for outcomes with ≥ 10 included studies using funnel plot visualisation and Egger's regression test for small‐study effects. For outcomes with < 10 studies, publication bias was not evaluated due to limited power of the available statistical tests.

## 3 Results

### 3.1 Search Results and Patient Characteristics

In our meta‐analysis, we identified 10 trials that met our inclusion criteria from a total of initial 2607 results [16, 17, 18, 19, 20, 21, 22, 23, 24, 25]. The PRISMA flowchart (Figure S1) summarises the search and trials selection. The included studies represent a diverse and robust sample across multiple countries, settings and trial design. The sample size ranged from a minimum of 24 to a maximum of 319 participants with a total of 786 patients (*n* = 436 in the closed‐loop AID arm; *n* = 350 in the conventional insulin delivery arm). Most of the studies evaluated closed‐loop insulin delivery systems, including MPC algorithms and commercial hybrid systems like Control IQ and DBLG1. The duration of intervention ranged from 24 h to 6 months and outcomes were assessed across overall, daytime and overnight periods. The baseline characteristics of patients for each individual trial are presented in Table 1. All the trials indicated a low risk of bias. (Figure S2).

**TABLE 1:** Baseline characteristics of included studies.

Study ID	Country	RCT design		Setting	Comparison	Sample size (*n*)		Device/intervention	Duration	Outcome periods
Thabit et al. (2015)	UK	Parallel	Single‐center	Inpatient	Conventional subcutaneous insulin delivery	20	20	MPC algorithm	72 h	Overall, daytime, overnight
Bally et al. (2019)	UK & Switzerland	Parallel	Multi‐center	Inpatient	Conventional insulin therapy	70	66	MPC algorithm	≤ 15 days or discharge	Overall, daytime, overnight
Boughton et al. (2019)	UK & Switzerland	Parallel	Multi‐center	Inpatient	Conventional insulin therapy	21	22	MPC algorithm	≤ 15 days or discharge	Overall, daytime, overnight
Herzig et al. (2019)	Switzerland	Parallel	Single‐center	Inpatient	Conventional insulin therapy	22	22	Cambridge adaptive MPC	≤ 20 days or discharge	Overall only
Boughton et al. (2020)	UK & Switzerland	Crossover	Multi‐center	Outpatient	Standard insulin therapy	26	26	Cambridge adaptive MPC	20 days	Overall only
Daly et al. (2020)	UK	Crossover	Single‐center	Outpatient	Standard insulin therapy	26	25	Cambridge adaptive MPC	8 weeks	Overall, daytime, overnight
Kumareswaran et al. (2019)	UK	Crossover	Single‐center	Inpatient	Glucose‐lowering medications	12	12	MPC algorithm	24 h	Overall and overnight
Borel et al. (2024)	France	Crossover	Multi‐center	Outpatient	Conventional subcutaneous insulin delivery + CGM	17		DBLG1 Hybrid Closed‐Loop	6 weeks	Overall, daytime, overnight
Kudva et al. (2025)	USA & Canada	Parallel	Multi‐center	Outpatient	CGM + standard insulin therapy	215	104	Tandem t:slim X2 with Control‐IQ++ Dexcom G6	13 weeks	Overall, daytime, overnight
Reznik et al. (2024)	France	Parallel	Multi‐center	Outpatient	MDI + home health care support	15	15	Tandem t:slim X2 with Control‐IQ+ Dexcom G6	12 weeks	Overall, daytime, overnight

### 3.2 Risk of Publication Bias

Publication bias among the included studies using reporting time in normoglycemic and hyperglycemic range was evaluated by Egger's test with a P‐value of 0.210 and 0.266, respectively (Figures S19 and S20), indicating the absence of publication bias.

### 3.3 Time Spent Near Normoglycemic Range

**FIGURE 1:**
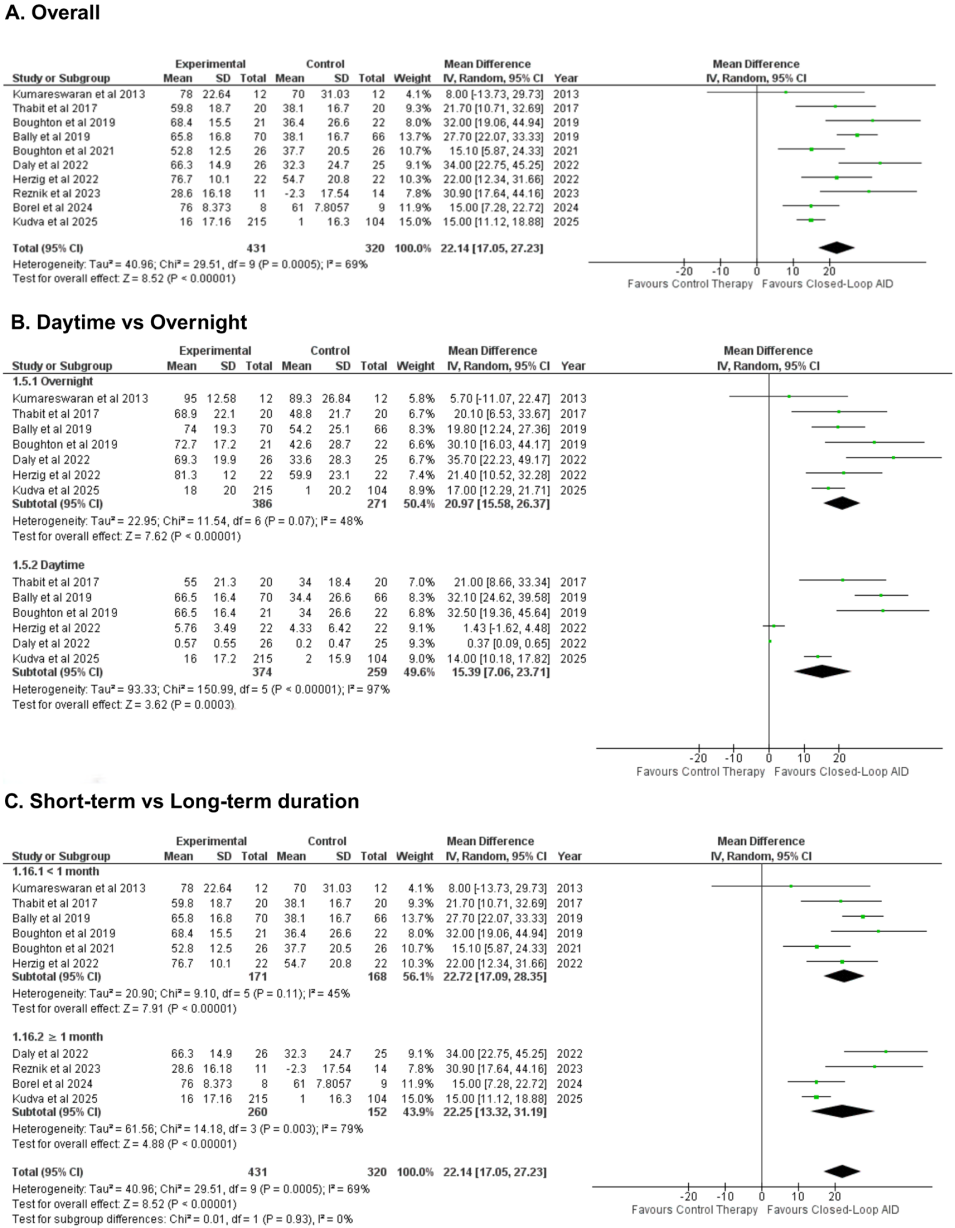
Pooled results for time spent in the near normoglycemic range.

Time spent near the normoglycemic range (in percentage change from baseline) was reported in 10 studies. Meta analysis of these studies showed that closed‐loop AID led to a significantly increased time spent in the normoglycemic range as compared to patients receiving conventional insulin delivery, corresponding to approximately 319 min/day more within range (WMD: 22.14% [17.05, 27.23]; P < 0.00001; *I* ^2^ = 69%) (Figure 1A). Subgroup analysis was conducted that did not show any significant modifications between daytime and overnight groups (WMD: 15.39% [7.06, 23.71], WMD: 20.97 [15.58, 26.37], respectively; Figure 1B), short‐term and long‐term duration (P‐interaction: 0.93; Figure 1C), or inpatient and outpatient settings (P‐interaction: 0.31; Figure S6). Sensitivity analysis by removing Borel et al. 2024 and Kudva et al. 2025 reduced heterogeneity, and the effect size remained consistent (WMD: 24.86% [19.80, 29.91];≈358 min/day; P < 0.00001; *I* ^2^ = 45%) (Figure S14).

### 3.4 Time Spent in Hypoglycemia

**FIGURE 2:**
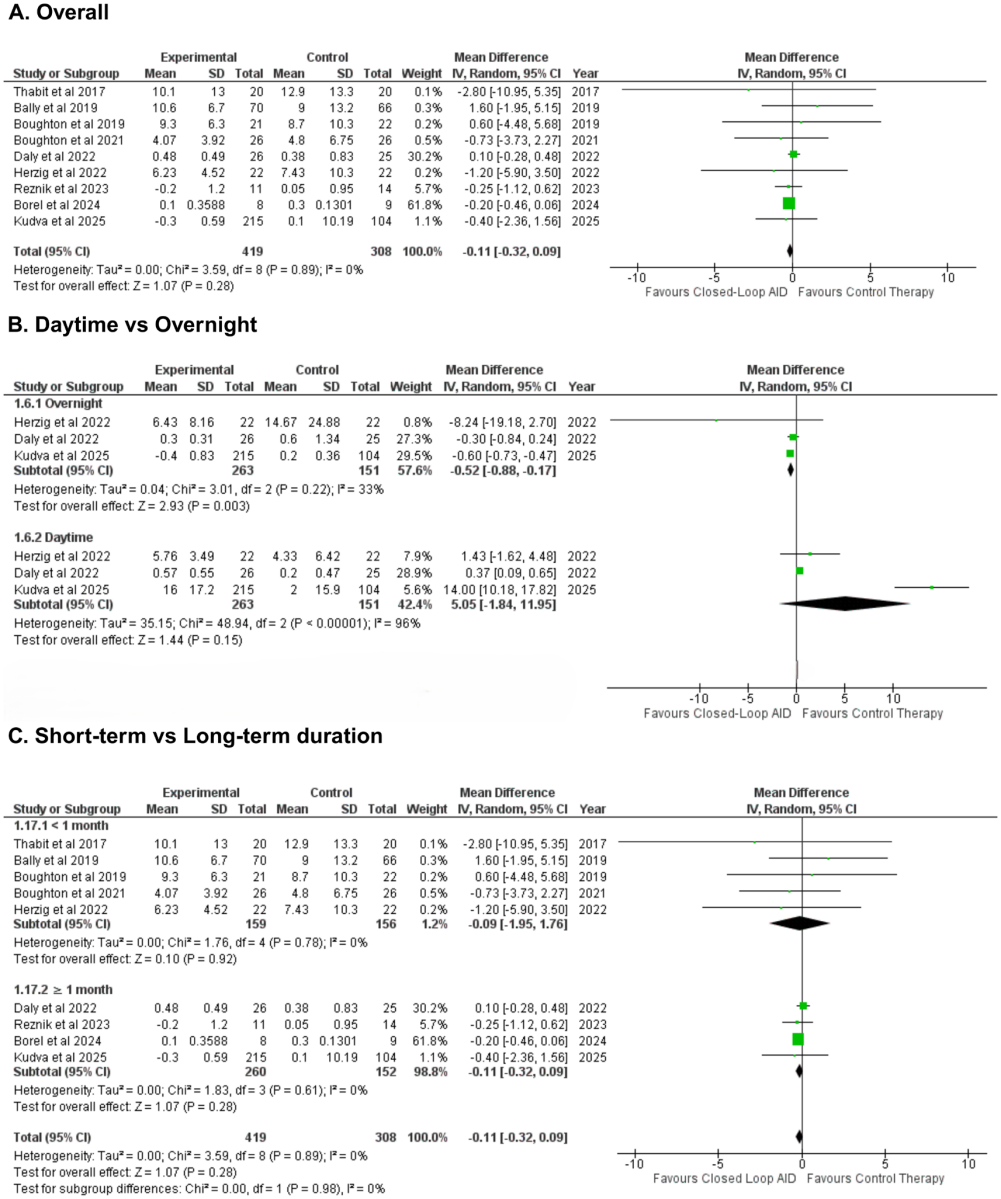
Pooled results for time spent in the near hypoglycemic range.

Nine studies reported time spent in the hypoglycemic state (in percentage change from baseline). Meta‐analysis indicated that patients receiving insulin via closed‐loop AID spent approximately 1.6 min less per day in hypoglycemia compared with those receiving conventional insulin delivery, a difference that was not statistically significant (WMD: −0.11% [−0.32, 0.09]; P = 0.28; *I* ^2^ = 0%) (Figure 2A). Subgroup analysis did not report a statistically significant difference based on measurements taken for daytime versus overnight (WMD: 5.05% [−1.84, 11.95], WMD: −0.52% [−0.88, −0.17], respectively; Figure 2B), short‐term and long‐term duration groups (P‐interaction: 0.98; Figure 2C) or care setting (P‐interaction = 0.73; Figure S7).

### 3.5 Time Spent in Hyperglycemia

**FIGURE 3:**
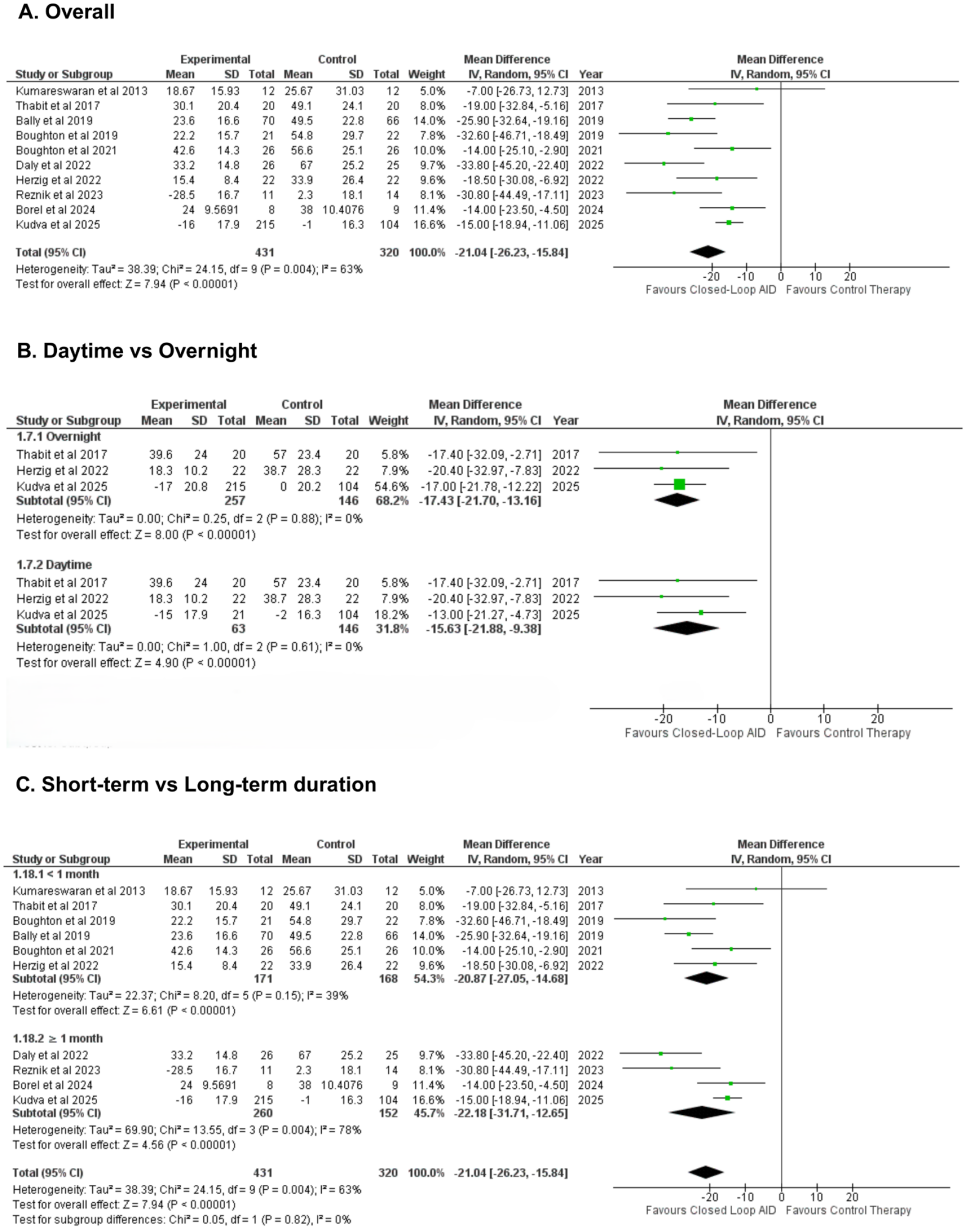
Pooled results for time spent in the near hyperglycemic range.

Time spent in hyperglycemic state (in percentage change from baseline) was reported in 10 studies. Closed‐loop AID resulted in patients spending approximately 303 min (about 5 h) less per day in the hyperglycemic range compared with conventional insulin delivery, demonstrating a significant reduction in hyperglycemia (WMD: −21.04% [−26.23, −15.84]; P 0.00001; *I* ^2^ = 63%) (Figure 3A). Subgroup analysis was conducted resulting in a non‐significant difference based on measurements taken, at daytime vs. overnight (WMD: −15.63% [−21.88, −9.38], WMD: −17.43% [−21.70, −13.16], respectively; Figure 3B), at short‐term vs. long‐term duration (P‐interaction: 0.82; Figure 3C), or inpatient vs. outpatient groups (‐interaction: 0.68; Figure S8). Sensitivity analysis was conducted by leaving out Borel et al. 2024 and Kudva et al. 2025, which reduced heterogeneity, while the effect size remained consistent (WMD: −23.60% [−29.33, −17.87]; P < 0.00001; *I* ^2^ = 46%) (Figure S15).

### 3.6 Glycated Haemoglobin Levels

**FIGURE 4:**
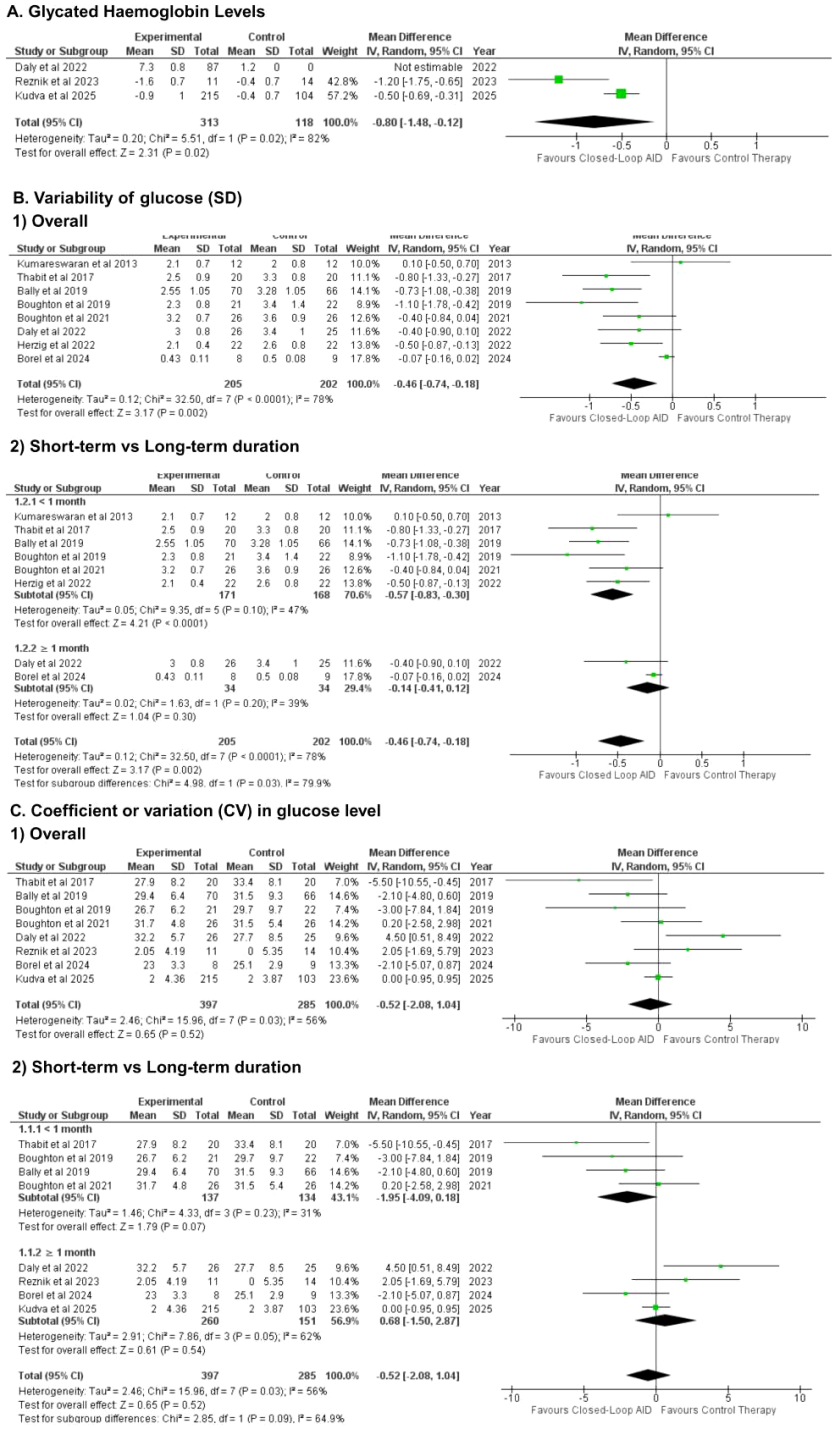
Pooled results for glycated haemoglobin, variability of glucose (SD), and coefficient of variation (CV) in glucose level.

Change in percentage glycated haemoglobin levels (HbA1c) was reported in 3 studies. Meta analysis of the studies showed statistically significant reduction in percentage glycated haemoglobin levels in administered insulin via closed‐loop AID as compared to the patients receiving insulin via the conventional method (WMD: −0.80% [−1.48, −0.12]; P = 0.02; *I* ^2^ = 82%) (Figure 4A).

### 3.7 Variability of Glucose

The variability of glucose (SD) was also evaluated via meta‐analysis. A statistically significant favourable effect on variability was highlighted in patients obtaining insulin via closed‐loop AID compared to the control group (WMD: −0.46 [−0.74, −0.18]; P = 0.002; *I* ^2^ = 78%) (Figure 4B). Subgroup analysis between durations demonstrated a significant difference, favouring short‐term duration (P‐interaction: 0.03; Figure 4B), including borderline significance favouring inpatient setting (P‐interaction: 0.05; Figure S9). The heterogeneity reduced, while the effect size remained consistent by removing Borel et al. 2024 (WMD: −0.54 [−0.71,−0.28]; P < 0.00001; *I* ^2^ = 38%) when sensitivity analysis was conducted (Figure S16).

### 3.8 Coefficient of Variation in Glucose Level

The coefficient of variation (CV) in glucose level (in percentage) was assessed in our meta analysis. The difference in CV in glucose level between the patients receiving insulin via closed‐loop AID and the control therapy group was statistically insignificant. (WMD: −0.52% [−2.08, −1.04]; P = 0.52; *I* ^2^ = 56%) (Figure 4C). Subgroup analysis revealed no statistically significant difference between short‐term and long‐term measurements (P‐interaction: 0.09; Figure 4C), though a significant improvement was observed in the inpatient subgroup (P‐interaction: 0.01; Figure S10). Upon omitting Daly et al. 2022, the heterogeneity reduced while the effect size remained consistent (WMD: −0.93% [−2.31, 0.46]; P = 0.19; *I* ^2^ = 42%) (Figure S17).

### 3.9 Total Daily Insulin

The change in total daily insulin levels (IU/day) was reported in 8 studies. Meta analysis of the studies did not show a statistically significant difference between the two groups (WMD: 2.08 [−5.67, 9.84]; P = 0.60; *I* ^2^ = 47%) (Figure S3). No significant differences were detected between short‐ and long‐term groups in the subgroup analysis (P‐interaction: 0.48; Figure S3).

### 3.10 Mean Glucose Levels

**FIGURE 5:**
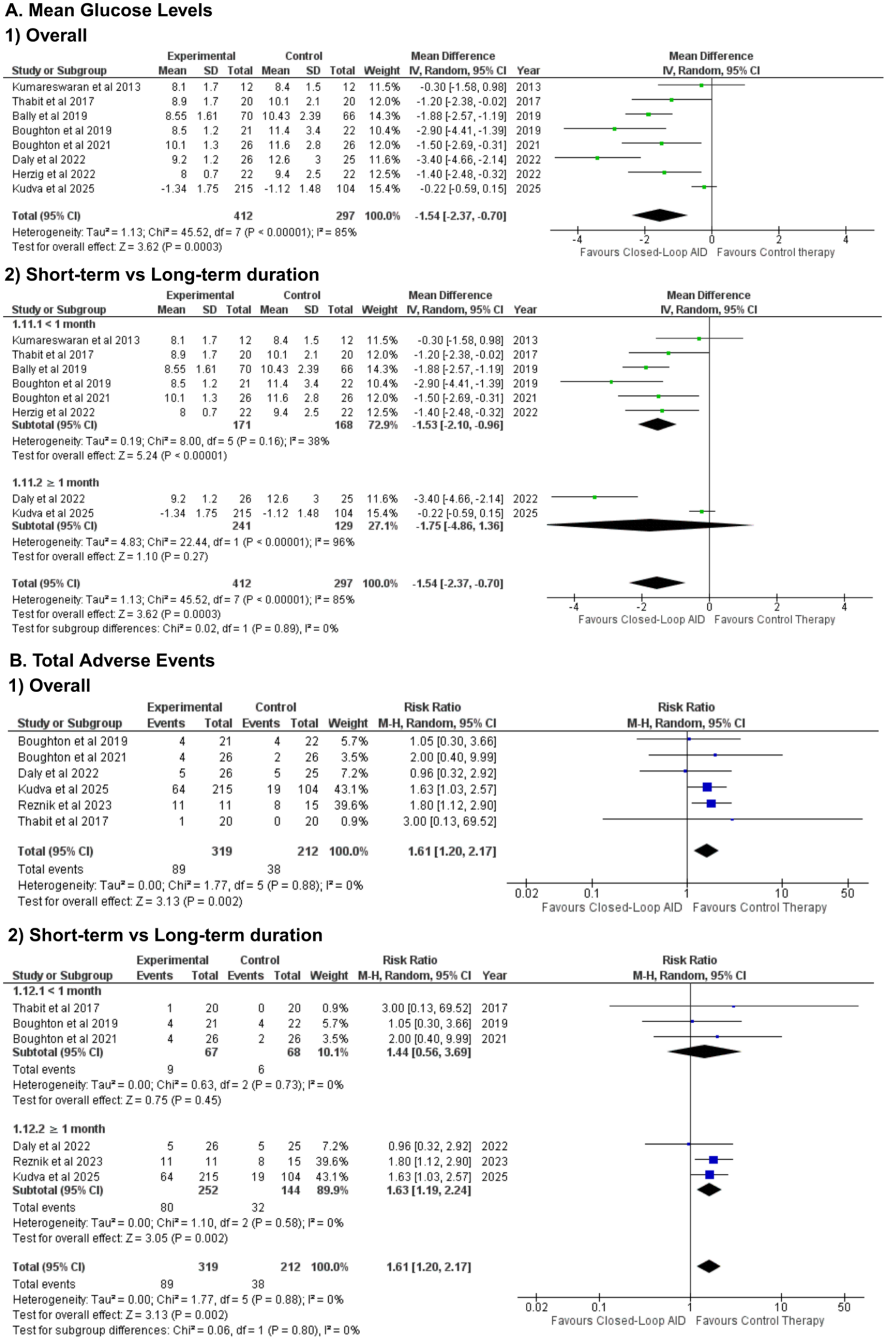
Pooled results for mean glucose levels and total adverse events.

The mean glucose levels (mmol/L) were reported in 8 studies. We evaluated that the mean glucose levels significantly reduced in the patients who were administered with insulin via the closed‐loop AID as compared to the control group (WMD: −1.54 mmol/L [−2.37, −0.70]; P = 0.0003; *I* ^2^ = 85%) (Figure 5A). Significant effect modification was observed between the measurements taken for short‐term and long‐term duration subgroups (P‐interaction: 0.02; Figure 5A). No significant differences were identified between inpatient and outpatient settings (P‐interaction: 0.91; Figure S11). Sensitivity analysis by omitting Daly et al. 2022 and Kudva et al. 2025 reduced the heterogeneity, while the effect size remained consistent (WMD: −1.53 mmol/L [−2.10, −0.96]; P < 0.00001; *I* ^2^ = 38%) (Figure S18).

### 3.11 Total Adverse Events

Total adverse events were reported in 6 studies. Meta analysis of these studies revealed that the closed‐loop insulation delivery method demonstrated a statistically stronger effect on the outcome, putting the experimental group at a higher risk of developing adverse events (RR: 1.61 [1.20, −2.17]; P = 0.002; *I* ^2^ = 0%) (Figure 5B). No significant subgroup differences were observed for duration (P‐interaction = 0.80; Figure 5C) or care setting (P‐interaction = 0.28; Figure S12).

### 3.12 Serious Adverse Events

The number of patients developing serious adverse events was reported in 8 studies. Meta analysis of these studies demonstrated that there was no significant difference between the two groups (RR: 1.61 [0.83, 3.13]; P = 0.16; *I* ^2^ = 10%) (Figure S4). Subgroup analysis revealed no statistically significant difference between short‐term and long‐term measurements (P‐interaction: 0.26; Figure S4) or between care setting (P‐interaction: 0.16; Figure S13).

### 3.13 Severe Hypoglycemic Event

Eight studies documented the number of patients who experienced severe hypoglycemia. Meta analysis of these studies showed no significant difference for developing severe hypoglycemia between the two groups (OR: 2.13 [0.22, 20.83]; P = 0.52; *I* ^2^ = 0%) (Figure S5). The subgroup analysis showed comparable results between short‐term and long‐term groups, with no statistical significance (P‐interaction: 0.74; Figure S5).

## 4 Discussion

This meta‐analysis, comprising 10 RCTs with a total of 786 patients, assessed the efficacy of closed‐loop AID compared to standard insulin therapy in patients with T2DM. Our findings revealed that closed‐loop AID significantly increased the time patients spent in the target normoglycemic range, both overall and during diurnal and overnight periods. Additionally, there was a significant reduction in time spent in the hyperglycemic range across all time periods. Closed‐loop AID also led to a significant improvement in mean glucose levels and significantly reduced glucose variability, as reflected by a lower standard deviation of glucose readings. Furthermore, the use of closed‐loop AID was associated with significant reductions in HbA1c, indicating improved long‐term glycemic control compared to conventional therapy. Although the occurrence of adverse events was reported to be significantly higher with closed‐loop AID, the absence of a significant increase in serious adverse events highlighted the manageable safety profile of closed‐loop AID. Overall, these findings underscore the promising potential of closed‐loop AID in enhancing glycemic control in patients with T2DM when compared to standard insulin regimens.

The previous meta‐analysis by Amer et al. provided valuable insights into the effectiveness of closed‐loop AID systems but had certain limitations [10], including a relatively limited sample size that may have produced unrealistically narrow confidence intervals, thereby reducing the reliability of its findings. In contrast, our meta‐analysis incorporated recent data from three additional major trials, nearly doubling the overall sample size and enhancing the robustness and generalizability of the results. Moreover, we evaluated HbA1c for the first time in this context, offering novel evidence on the long‐term glycemic benefits of closed‐loop AID in patients with T2DM. We also conducted subgroup analyses stratified by follow‐up duration across all glycemic outcomes, further strengthening the credibility of our conclusions. Consistent with Amer et al., our findings reaffirm the superior efficacy of closed‐loop AID systems over conventional insulin delivery in improving glycemic outcomes among patients with T2DM, demonstrating significant increases in time within the normoglycemic range and reductions in hyperglycemia. However, unlike the prior meta‐analysis, we observed a significantly higher incidence of total adverse events with closed‐loop systems, though these were predominantly device‐related and not associated with an increase in serious adverse events. Furthermore, our results align with those of Wang et al. [26], who reported that AI‐driven closed‐loop systems were significantly associated with greater time in target glucose range and reduced hyperglycemia compared to traditional diabetes therapies, collectively underscoring the potential of AI‐driven approaches to enhance glycemic control and advance future diabetes management.

Our meta‐analysis highlights the significant glycemic benefits of closed‐loop AID systems in patients with T2DM, demonstrating substantial improvements in time spent in the normoglycemic range and reductions in hyperglycemia and mean glucose levels. These effects were consistent across daytime and overnight measurements and across both short‐ and long‐term durations, suggesting the robustness and generalizability of the intervention. The observed advantages of automated closed loop systems over conventional therapy can be explained by the underlying mechanism by which closed‐loop AID systems operate. AID systems incorporate a CGM that utilizes a subcutaneous sensor to monitor interstitial glucose levels every 1–5 min, while conventional insulin therapy involves manual insulin delivery through injections or pumps [27]. This real‐time data enables the AID system to promptly adjust insulin delivery, addressing glycemic variability, a key target alongside HbA1c for minimizing diabetes complications, which is not accurately captured by intermittent self‐monitoring of blood glucose readings [28, 29]. Moreover, insulin pumps mimic physiological pancreatic insulin delivery by providing continuous basal insulin and precise bolus dosing, resulting in a more stable blood glucose response compared to insulin injections [30, 31]. Additionally, insulin pumps offer the ability to program multiple basal rate profiles, enabling patients to tailor insulin delivery to variations in daily or weekly routines [32]. This flexibility allows for precise adjustments in response to factors such as physical activity or stress, adaptations that are not achievable with fixed dose insulin injection regimens [33]. These capabilities may help explain the increased time spent within the target glucose range observed with closed‐loop AID systems compared to conventional treatment.

This study demonstrated that closed‐loop AID was associated with improved HbA1c levels, suggesting a greater potential for reducing diabetes‐related complications in patients with T2DM. Given the well‐established association between elevated HbA1c and the risk of diabetic retinopathy, nephropathy, and other microvascular complications, as supported by previous research [22]. Improvements in glucose variability, as reflected by reductions in standard deviation and mean glucose levels, were particularly evident in long‐term user subgroups, indicating that extended use of closed‐loop AID may yield greater glycemic benefits. Notably, however, despite achieving better overall glycemic control, the system did not significantly reduce time spent in hypoglycemia or the incidence of severe hypoglycemic events. This highlights potential limitations in the safety thresholds or algorithmic responsiveness of current AID technologies. Additionally, while the observed increase in total adverse events was not accompanied by a rise in serious complications, it raises concerns, likely attributable to device‐related or minor issues. The definition of adverse events was consistent across studies, in accordance with ISO 14155:3.2, which includes both clinical and device‐related events. Although most adverse events were associated with the investigational devices, Kudva et al. reported a higher incidence of non‐severe hypoglycemia in the closed‐loop AID group [22]. Thabit et al. documented a single adverse event unrelated to the study devices, an episode of gastrointestinal bleeding in the closed‐loop group, with no serious adverse events occurring in either group [25]. Reznik et al. found a higher proportion of diabetes‐related adverse events linked to closed‐loop systems [24]. Overall, adverse events were predominantly device‐related. These findings suggest that although AID systems can effectively automate routine insulin adjustments, they may still require careful oversight, particularly in high‐risk scenarios where clinical judgement and patient experience remain crucial.

Despite overall consistency of our analysis, heterogeneity was noted in some outcomes, stemming from variations in study design, patient characteristics, monitoring technologies, and clinical settings. The studies varied significantly in follow‐up periods, ranging from as short as 24 h in Kumareswaran et al. [23] to 13 weeks in Kudva et al. [22] and Borel et al. [17] Short‐term inpatient studies captured immediate glycemic effects but may have missed behavioural adaptations such as changes in diet, exercise, and familiarity with device use that evolved during longer outpatient follow‐ups. These long‐term behavioural changes could play a major role in enhancing glycemic control, contributing to variability across studies [34, 35]. However, subgroup analyses stratified by follow‐up duration for glycemic ranges revealed no significant differences in treatment effect, reinforcing the consistency and robustness of our pooled findings despite these variations. Trial setting was a further contributor to heterogeneity. Inpatient studies such Bally et al. [16], Herzig et al. [21], Thabit et al. [25] and Kumareswaran et al. [23], benefit from closely monitored, standardised conditions, where trained healthcare workers manage device setup and troubleshoot issues swiftly, ensuring optimal performance of the system. Whereas outpatient trials such as Kudva et al. [22], Reznik et al. [24], Daly et al. [20] and Borel et al. [17], relied mainly on patients' management, potentially increasing user variability and error. Studies also incorporated a range of CGM systems which may potentially be a reason for technical heterogeneity. Qualitatively, both fully closed‐loop and hybrid closed‐loop systems demonstrated improvements in glycemic outcomes compared with standard care. The direction and magnitude of effect were broadly similar across algorithm types, suggesting that the observed benefits were not driven solely by fully closed‐loop systems. However, the small number of hybrid trials limits definitive interpretation. Furthermore, bally et al. [16] had patients with renal impairment requiring dialysis over the course of the trial. This comorbid status can potentially affect the performance of closed‐loop AID systems, as glucose homeostasis is significantly altered in individuals with renal complications [36, 37].

Future research should focus on conducting long‐duration trials with multiple follow‐up points to effectively evaluate both short‐term and long‐term efficacy of closed‐loop AID systems. Furthermore, more trials should prioritise the reporting of race‐specific data to enable clinicians to assess differential responses across diverse ethnic populations, thereby allowing clinicians to make more personalised treatment strategies. Conducting studies across varied geographic regions is also encouraged to reduce population bias and enhance the generalizability of findings. Trials exploring the co‐use of AID systems with other glucose‐lowering agents, such as GLP‐1 receptor agonists or SGLT2 inhibitors, would provide insights into potential benefits of combination therapies. Finally, comparisons of different AID systems and CGM technologies are necessary to identify the most effective devices for optimising clinical outcomes in T2DM.

### 4.1 Limitations

A few limitations need to be addressed. First, our meta‐analysis included a relatively small number of trials and participants for long follow‐up durations, limiting the reliability of data on outcomes such as HbA1c levels and long‐term safety of closed loop systems. Second, the types of glucose monitoring devices and insulin pumps varied between studies, introducing technical variability. Third, diabetes duration before inclusion was not consistent among the included RCTs, potentially affecting responsiveness to AID. Fourth, patients from limited geographic locations were included, mainly from the UK and Switzerland, thereby limiting generalizability to all global populations.

## 5 Conclusion

This meta‐analysis of 786 patients with T2DM demonstrates that closed‐loop AID systems significantly increase time spent in the near‐normoglycemic range while reducing time in hyperglycemia, without elevating the risk of serious adverse events such as severe hypoglycemia. These findings support the clinical utility of closed‐loop AID in the management of T2DM over conventional insulin and underscore the need for further research with extended follow‐up to guide broader adoption in routine care.

## Author Contributions

**Dua Ali:** conceptualization, project administration, writing – original draft, writing – review and editing. **Abdur Rafay Bilal:** writing – original draft, writing – review and editing, investigation. **S. M. Washaqul Arfin:** writing – original draft, formal analysis, data curation; **Maryam Sajid:** visualisation, writing – original draft, writing – review and editing; **Shaheer Qureshi:** methodology, writing – original draft, writing – review and editing; **Raheel Ahmed:** validation, supervision; **Hasibullah Aminpoor:** writing – review and editing, supervision, validation; **Saad Ahmed Waqas:** validation, writing – review and editing. All authors read and approved the final manuscript.

## Funding

The authors have nothing to report.

## Ethics Statement

The authors have nothing to report.

## Conflicts of Interest

The authors declare no conflicts of interest.

## Supporting information


**Appendix S1:** edm270178‐sup‐0001‐AppendixS1.docx.
**Table S1:** Detailed search string for extracted studies form databases.
**Figure S1:** PRISMA flowchart.
**Figure S2:** Risk of bias assessment of the included RCTs.
**Figure S3:** Total Daily Insulin.
**Figure S4:** Serious Adverse Events.
**Figure S5:** Severe Hypoglycemic event.
**Figure S6:** Time spent in normoglycemic range.
**Figure S7:** Time spent in hypoglycemic range.
**Figure S8:** Time spent in hyperglycemic range.
**Figure S9:** Variability of glucose (SD).
**Figure S10:** Coefficient of variation (CV) of glucose.
**Figure S11:** Mean glucose levels.
**Figure S12:** Total adverse events.
**Figure S13:** Serious adverse events.
**Figure S14:** Time Spent Near Normoglycemic Range after exclusion of Borel et al. 2024 and Kudva et al. 2025.
**Figure S15:** Time Spent Near Hyperglycemic Range after exclusion of Borel et al. 2024 and Kudva et al. 2025.
**Figure S16:** Variability of glucose (SD) after exclusion of Borel et al. 2024.
**Figure S17:** Coefficient or variation (CV) in glucose level after exclusion of Daly et al. 2022.
**Figure S18:** Mean glucose levels after exclusion of omitting Daly et al. 2022 and Kudva et al. 2025.
**Figure S19:** Publication bias measured by Egger's test on the time spent in normoglycemic range.
**Figure S20:** Publication bias measured by Egger's test on the time spent in hyperglycemic range.

## Data Availability

The data supporting the findings of this study were obtained from published Randomised Controlled Trials. The data and materials used were publicly available.

## References

[1] International Diabetes Federation , “Facts and Figures,” (2025), https://idf.org/about‐diabetes/diabetes‐facts‐figures/.

[2] “Diabetes,” (2025), https://www.who.int/news‐room/fact‐sheets/detail/diabetes.

[3] M. Roden and G. I. Shulman , “The Integrative Biology of Type 2 Diabetes,” Nature 576, no. 7785 (2019): 51–60, 10.1038/s41586-019-1797-8. 31802013

[4] W. H. Polonsky and R. R. Henry , “Poor Medication Adherence in Type 2 Diabetes: Recognizing the Scope of the Problem and Its Key Contributors,” Patient Preference and Adherence 10 (2016): 1299–1307, 10.2147/PPA.S106821. 27524885 PMC4966497

[5] “Insulin‐Pharmacology, Therapeutic Regimens and Principles of Intensive Insulin Therapy – Endotext—NCBI Bookshelf,” (2025), https://www.ncbi.nlm.nih.gov/books/NBK278938/.

[6] J. L. Sherr , L. Heinemann , G. A. Fleming , et al., “Automated Insulin Delivery: Benefits, Challenges, and Recommendations. A Consensus Report of the Joint Diabetes Technology Working Group of the European Association for the Study of Diabetes and the American Diabetes Association,” Diabetes Care 45, no. 12 (2022): 3058–3074, 10.2337/dci22-0018. 36202061

[7] A. Stahl‐Pehe , S. Schlesinger , O. Kuss , et al., “Efficacy of Automated Insulin Delivery (AID) Systems in Type 1 Diabetes: Protocol of a Systematic Review and Network Meta‐Analysis of Outpatient Randomised Controlled Trials,” BMJ Open 13, no. 10 (2023): e074317, 10.1136/bmjopen-2023-074317. PMC10565260 37816564

[8] “Type 1 vs Type 2 Diabetes|UVA Health,” (2025), https://uvahealth.com/services/diabetes‐care/types.

[9] M. Gallo , E. Mannucci , S. D. Cosmo , et al., “Algorithms for Personalized Therapy of Type 2 Diabetes: Results of a Web‐Based International Survey,” BMJ Open Diabetes Research & Care 3, no. 1 (2015): e000109, 10.1136/bmjdrc-2015-000109. PMC4537916 26301097

[10] B. E. Amer , Y. E. Amer , A. M. Abozaid , et al., “Does Fully Closed‐Loop Automated Insulin Delivery Improve Glycaemic Control in Patients With Type 2 Diabetes? A Meta‐Analysis of Randomized Controlled Trials,” Diabetic Medicine 41, no. 1 (2024): e15196. 37567739 10.1111/dme.15196

[11] A. Liberati , D. G. Altman , J. Tetzlaff , et al., “The PRISMA Statement for Reporting Systematic Reviews and Meta‐Analyses of Studies That Evaluate Healthcare Interventions: Explanation and Elaboration,” British Medical Journal 339 (2009): b2700, 10.1136/bmj.b2700. 19622552 PMC2714672

[12] J. P. T. Higgins , J. Thomas , J. Chandler , et al., eds., Cochrane Handbook for Systematic Reviews of Interventions, 1st ed. (Wiley, 2019), 10.1002/9781119536604.

[13] M. Ouzzani , H. Hammady , Z. Fedorowicz , and A. Elmagarmid , “Rayyan—A Web and Mobile App for Systematic Reviews,” Systematic Reviews 5, no. 1 (2016): 210, 10.1186/s13643-016-0384-4. 27919275 PMC5139140

[14] J. P. T. Higgins , D. G. Altman , P. C. Gøtzsche , et al., “The Cochrane Collaboration's Tool for Assessing Risk of Bias in Randomised Trials,” BMJ 343 (2011): d5928, 10.1136/bmj.d5928. 22008217 PMC3196245

[15] “Measuring Inconsistency in Meta‐Analyses PubMed,” (2025), https://pubmed.ncbi.nlm.nih.gov/12958120/.

[16] L. Bally , P. Gubler , H. Thabit , et al., “Fully Closed‐Loop Insulin Delivery Improves Glucose Control of Inpatients With Type 2 Diabetes Receiving Hemodialysis,” Kidney International 96, no. 3 (2019): 593–596, 10.1016/j.kint.2019.03.006. 31133457

[17] A. L. Borel , S. Lablanche , C. Waterlot , et al., “Closed‐Loop Insulin Therapy for People With Type 2 Diabetes Treated With an Insulin Pump: A 12‐Week Multicenter, Open‐Label Randomized, Controlled, Crossover Trial,” Diabetes Care 47, no. 10 (2024): 1778–1786, 10.2337/dc24-0623. 39106206 PMC11417293

[18] C. K. Boughton , L. Bally , F. Martignoni , et al., “Fully Closed‐Loop Insulin Delivery in Inpatients Receiving Nutritional Support: A Two‐Centre, Open‐Label, Randomised Controlled Trial,” Lancet Diabetes & Endocrinology 7, no. 5 (2019): 368–377, 10.1016/S2213-8587(19)30061-0. 30935872 PMC6467839

[19] C. K. Boughton , A. Tripyla , S. Hartnell , et al., “Fully Automated Closed‐Loop Glucose Control Compared With Standard Insulin Therapy in Adults With Type 2 Diabetes Requiring Dialysis: An Open‐Label, Randomized Crossover Trial,” Nature Medicine 27, no. 8 (2021): 1471–1476, 10.1038/s41591-021-01453-z. PMC8363503 34349267

[20] A. B. Daly , C. K. Boughton , M. Nwokolo , et al., “Fully Automated Closed‐Loop Insulin Delivery in Adults With Type 2 Diabetes: An Open‐Label, Single‐Center, Randomized Crossover Trial,” Nature Medicine 29, no. 1 (2023): 203–208, 10.1038/s41591-022-02144-z. PMC9873557 36631592

[21] D. Herzig , S. Suhner , J. Roos , et al., “Perioperative Fully Closed‐Loop Insulin Delivery in Patients Undergoing Elective Surgery: An Open‐Label, Randomized Controlled Trial,” Diabetes Care 45, no. 9 (2022): 2076–2083, 10.2337/dc22-0438. 35880252

[22] Y. C. Kudva , D. Raghinaru , J. W. Lum , et al., “A Randomized Trial of Automated Insulin Delivery in Type 2 Diabetes,” New England Journal of Medicine 392, no. 18 (2025): 1801–1812, 10.1056/NEJMoa2415948. 40105270

[23] K. Kumareswaran , H. Thabit , L. Leelarathna , et al., “Feasibility of Closed‐Loop Insulin Delivery in Type 2 Diabetes: A Randomized Controlled Study,” Diabetes Care 37, no. 5 (2014): 1198–1203, 10.2337/dc13-1030. 24026542

[24] Y. Reznik , M. Carvalho , S. Fendri , et al., “Should People With Type 2 Diabetes Treated by Multiple Daily Insulin Injections With Home Health Care Support Be Switched to Hybrid Closed‐Loop? The CLOSE AP+ Randomized Controlled Trial,” Diabetes, Obesity & Metabolism 26, no. 2 (2024): 622–630, 10.1111/dom.15351. 37921083

[25] H. Thabit , S. Hartnell , J. M. Allen , et al., “Closed‐Loop Insulin Delivery in Inpatients With Type 2 Diabetes: A Randomised, Parallel‐Group Trial,” Lancet Diabetes & Endocrinology 5, no. 2 (2017): 117–124, 10.1016/S2213-8587(16)30280-7. 27836235

[26] X. Wang , J. Si , Y. Li , et al., “Effectiveness and Safety of AI‐Driven Closed‐Loop Systems in Diabetes Management: A Systematic Review and Meta‐Analysis,” Diabetology and Metabolic Syndrome 17, no. 1 (2025): 238, 10.1186/s13098-025-01819-0. 40551160 PMC12183835

[27] “Continuous Glucose Monitoring—An Overview|ScienceDirect Topics,” (2025), https://www.sciencedirect.com/topics/nursing‐and‐health‐professions/continuous‐glucose‐monitoring.

[28] G. Cappon , M. Vettoretti , G. Sparacino , and A. Facchinetti , “Continuous Glucose Monitoring Sensors for Diabetes Management: A Review of Technologies and Applications,” Diabetes and Metabolism Journal 43, no. 4 (2019): 383–397, 10.4093/dmj.2019.0121. 31441246 PMC6712232

[29] I. B. Hirsch , “Glycemic Variability and Diabetes Complications: Does it Matter? Of Course it Does!,” Diabetes Care 38, no. 8 (2015): 1610–1614, 10.2337/dc14-2898. 26207054

[30] C. Berget , L. H. Messer , and G. P. Forlenza , “A Clinical Overview of Insulin Pump Therapy for the Management of Diabetes: Past, Present, and Future of Intensive Therapy,” Diabetes Spectrum: A Publication of the American Diabetes Association 32, no. 3 (2019): 194–204, 10.2337/ds18-0091. 31462873 PMC6695255

[31] Y. Reznik , O. Cohen , R. Aronson , et al., “Insulin Pump Treatment Compared With Multiple Daily Injections for Treatment of Type 2 Diabetes (OpT2mise): A Randomised Open‐Label Controlled Trial,” Lancet 384, no. 9950 (2014): 1265–1272, 10.1016/S0140-6736(14)61037-0. 24998009

[32] J. L. Selam , “Evolution of Diabetes Insulin Delivery Devices,” Journal of Diabetes Science and Technology 4, no. 3 (2010): 505–513, 10.1177/193229681000400302. 20513314 PMC2901025

[33] J. E. Yardley , K. E. Iscoe , R. J. Sigal , G. P. Kenny , B. A. Perkins , and M. C. Riddell , “Insulin Pump Therapy Is Associated With Less Post‐Exercise Hyperglycemia Than Multiple Daily Injections: An Observational Study of Physically Active Type 1 Diabetes Patients,” Diabetes Technology & Therapeutics 15, no. 1 (2013): 84–88, 10.1089/dia.2012.0168. 23216304

[34] S. Z. A. Shah , J. A. Karam , A. Zeb , et al., “Movement is Improvement: The Therapeutic Effects of Exercise and General Physical Activity on Glycemic Control in Patients With Type 2 Diabetes Mellitus: A Systematic Review and Meta‐Analysis of Randomized Controlled Trials,” Diabetes Therapy: Research, Treatment and Education of Diabetes and Related Disorders 12, no. 3 (2021): 707–732. 33547579 10.1007/s13300-021-01005-1 PMC7947168

[35] M. E. Al‐Adwi , Z. M. Al‐Haswsa , K. M. Alhmmadi , et al., “Effects of Different Diets on Glycemic Control Among Patients With Type 2 Diabetes: A Literature Review,” Nutrition and Health 29, no. 2 (2023): 215–221, 10.1177/02601060221112805. 35795964

[36] M. Guthoff , R. Wagner , D. Vosseler , et al., “Impact of End‐Stage Renal Disease on Glucose Metabolism—A Matched Cohort Analysis,” Nephrology, Dialysis, Transplantation 32, no. 4 (2017): 670–676, 10.1093/ndt/gfx018. 28407130

[37] R. J. Galindo , R. W. Beck , M. F. Scioscia , G. E. Umpierrez , and K. R. Tuttle , “Glycemic Monitoring and Management in Advanced Chronic Kidney Disease,” Endocrine Reviews 41, no. 5 (2020): 756–774, 10.1210/endrev/bnaa017. 32455432 PMC7366347

