# Supplementary material for: Closed‐Loop Automated Insulin Delivery in Patients With Type 2 Diabetes: A Meta‐Analysis of Randomised Controlled Trials
Source: Endocrinol Diabetes Metab. 2026 Mar 13;9(2):e70178. doi: 10.1002/edm2.70178 (PMC13093502; doi:10.1002/edm2.70178)

**Supplementary Appendix**

**Online supplementary material 1:** Search strategy, PRISMA flowchart and Risk of Bias Assessment

**Online supplementary material 2:** Forest Plots

**Online supplementary material 3:** Forest plots subgrouped by care setting

**Online supplementary material 4:** Sensitivity Analysis plots

**Online supplementary material 5:** Egger’s publication bias plots

**Online supplementary material 1:** Search strategy, PRISMA flowchart and Risk of Bias Assessment

**Table S1.** Detailed search string for extracted studies form databases.

| **Database** | **String** | **Results** |
| --- | --- | --- |
| **PubMed** | ("diabetes mellitus, type 2"[MeSH Terms] OR "type 2 diabetes mellitus"[All Fields] OR "type 2 diabetes"[All Fields] OR ("diabetes mellitus, type 2"[MeSH Terms] OR "type 2 diabetes mellitus"[All Fields]) OR "T2DM"[All Fields] OR ("diabete"[All Fields] OR "diabetes mellitus"[MeSH Terms] OR ("diabetes"[All Fields] AND "mellitus"[All Fields]) OR "diabetes mellitus"[All Fields] OR "diabetes"[All Fields] OR "diabetes insipidus"[MeSH Terms] OR ("diabetes"[All Fields] AND "insipidus"[All Fields]) OR "diabetes insipidus"[All Fields] OR "diabetic"[All Fields] OR "diabetics"[All Fields] OR "diabets"[All Fields])) AND (("closed-loop"[All Fields] AND ("insulin"[Supplementary Concept] OR "insulin"[All Fields] OR "insulin"[MeSH Terms] OR "insulin s"[All Fields] OR "insuline"[All Fields] OR "insulinic"[All Fields] OR "insulinization"[All Fields] OR "insulinized"[All Fields] OR "insulins"[MeSH Terms] OR "insulins"[All Fields]) AND ("deliveries"[All Fields] OR "delivery, obstetric"[MeSH Terms] OR ("delivery"[All Fields] AND "obstetric"[All Fields]) OR "obstetric delivery"[All Fields] OR "delivery"[All Fields])) OR (("automate"[All Fields] OR "automated"[All Fields] OR "automates"[All Fields] OR "automating"[All Fields] OR "automation"[MeSH Terms] OR "automation"[All Fields] OR "automations"[All Fields] OR "automation s"[All Fields]) AND ("insulin"[Supplementary Concept] OR "insulin"[All Fields] OR "insulin"[MeSH Terms] OR "insulin s"[All Fields] OR "insuline"[All Fields] OR "insulinic"[All Fields] OR "insulinization"[All Fields] OR "insulinized"[All Fields] OR "insulins"[MeSH Terms] OR "insulins"[All Fields]) AND ("deliveries"[All Fields] OR "delivery, obstetric"[MeSH Terms] OR ("delivery"[All Fields] AND "obstetric"[All Fields]) OR "obstetric delivery"[All Fields] OR "delivery"[All Fields])) OR ("pancreas, artificial"[MeSH Terms] OR ("pancreas"[All Fields] AND "artificial"[All Fields]) OR "artificial pancreas"[All Fields] OR ("artificial"[All Fields] AND "pancreas"[All Fields])) OR (("chimera"[MeSH Terms] OR "chimera"[All Fields] OR "hybrid"[All Fields] OR "hybrids"[All Fields] OR "hybrid s"[All Fields] OR "hybridation"[All Fields] OR "hybridisations"[All Fields] OR "hybridise"[All Fields] OR "hybridised"[All Fields] OR "hybridises"[All Fields] OR "hybridising"[All Fields] OR "hybridity"[All Fields] OR "hybridization, genetic"[MeSH Terms] OR ("hybridization"[All Fields] AND "genetic"[All Fields]) OR "genetic hybridization"[All Fields] OR "hybridisation"[All Fields] OR "hybridizations"[All Fields] OR "hybridize"[All Fields] OR "hybridized"[All Fields] OR "hybridizes"[All Fields] OR "hybridizing"[All Fields] OR "nucleic acid hybridization"[MeSH Terms] OR ("nucleic"[All Fields] AND "acid"[All Fields] AND "hybridization"[All Fields]) OR "nucleic acid hybridization"[All Fields] OR "hybridization"[All Fields]) AND ("close"[All Fields] OR "closed"[All Fields] OR "closely"[All Fields] OR "closeness"[All Fields] OR "closes"[All Fields] OR "closing"[All Fields] OR "closings"[All Fields]) AND "loop"[All Fields]) OR ("fully"[All Fields] AND ("close"[All Fields] OR "closed"[All Fields] OR "closely"[All Fields] OR "closeness"[All Fields] OR "closes"[All Fields] OR "closing"[All Fields] OR "closings"[All Fields]) AND "loop"[All Fields])) AND ("standard of care"[MeSH Terms] OR ("standard"[All Fields] AND "care"[All Fields]) OR "standard of care"[All Fields] OR ("standard"[All Fields] AND "therapy"[All Fields]) OR "standard therapy"[All Fields] OR (("conventional"[All Fields] OR "conventionals"[All Fields]) AND ("insulin"[Supplementary Concept] OR "insulin"[All Fields] OR "insulin"[MeSH Terms] OR "insulin s"[All Fields] OR "insuline"[All Fields] OR "insulinic"[All Fields] OR "insulinization"[All Fields] OR "insulinized"[All Fields] OR "insulins"[MeSH Terms] OR "insulins"[All Fields]) AND ("therapeutics"[MeSH Terms] OR "therapeutics"[All Fields] OR "therapies"[All Fields] OR "therapy"[MeSH Subheading] OR "therapy"[All Fields] OR "therapy s"[All Fields] OR "therapys"[All Fields])) OR (("multiple"[All Fields] OR "multiples"[All Fields]) AND ("dailies"[All Fields] OR "daily"[All Fields]) AND ("inject"[All Fields] OR "injectability"[All Fields] OR "injectant"[All Fields] OR "injectants"[All Fields] OR "injectate"[All Fields] OR "injectates"[All Fields] OR "injected"[All Fields] OR "injectible"[All Fields] OR "injectibles"[All Fields] OR "injecting"[All Fields] OR "injections"[MeSH Terms] OR "injections"[All Fields] OR "injectable"[All Fields] OR "injectables"[All Fields] OR "injection"[All Fields] OR "injects"[All Fields])) OR ("usual"[All Fields] AND "care"[All Fields])) | 914 |
| **Cochrane Library** | (type 2 diabetes OR type 2 diabetes mellitus OR T2DM OR diabetes) AND (closed-loop insulin delivery OR automated insulin delivery OR artificial pancreas OR hybrid closed loop OR fully closed loop) AND (standard therapy OR conventional insulin therapy OR multiple daily injections OR usual care) AND (glycemic control OR glycaemic control OR HbA1c OR glucose levels OR time in range) | 780 |
| **Scopus** | (type 2 diabetes OR type 2 diabetes mellitus OR T2DM OR diabetes) AND (closed-loop insulin delivery OR automated insulin delivery OR artificial pancreas OR hybrid closed loop OR fully closed loop) AND (standard therapy OR conventional insulin therapy OR multiple daily injections OR usual care) AND (glycemic control OR glycaemic control OR HbA1c OR glucose levels OR time in range) | 913 |

**Figure S1.** PRISMA flowchart

**Identification of studies via databases**

Records removed *before screening*:

Duplicate records removed
 (n = 1258)

Records identified from: Pubmed (n = 914), Scopus (n = 913), and Cochrane Library (n = 780)

(N= 2607)

**Identification**

Records screened in total.

(n = 1358)

Records excluded based on title and abstract.

(n = 1252)

**Screening**

Records excluded.

Irrelevant studies (n=40)

Irrelevant outcome (n=22)

Review studies (n=27)

Inappropriate comparison= (n=105)

Protocols (n=1)

Potentially relevant articles identified for full text review
(n =106)

**Included**

Studies included in qualitative- synthesis (n = 10)

**Figure S2:** Risk of bias assessment of the included RCTs.

1. Parallel RCTs


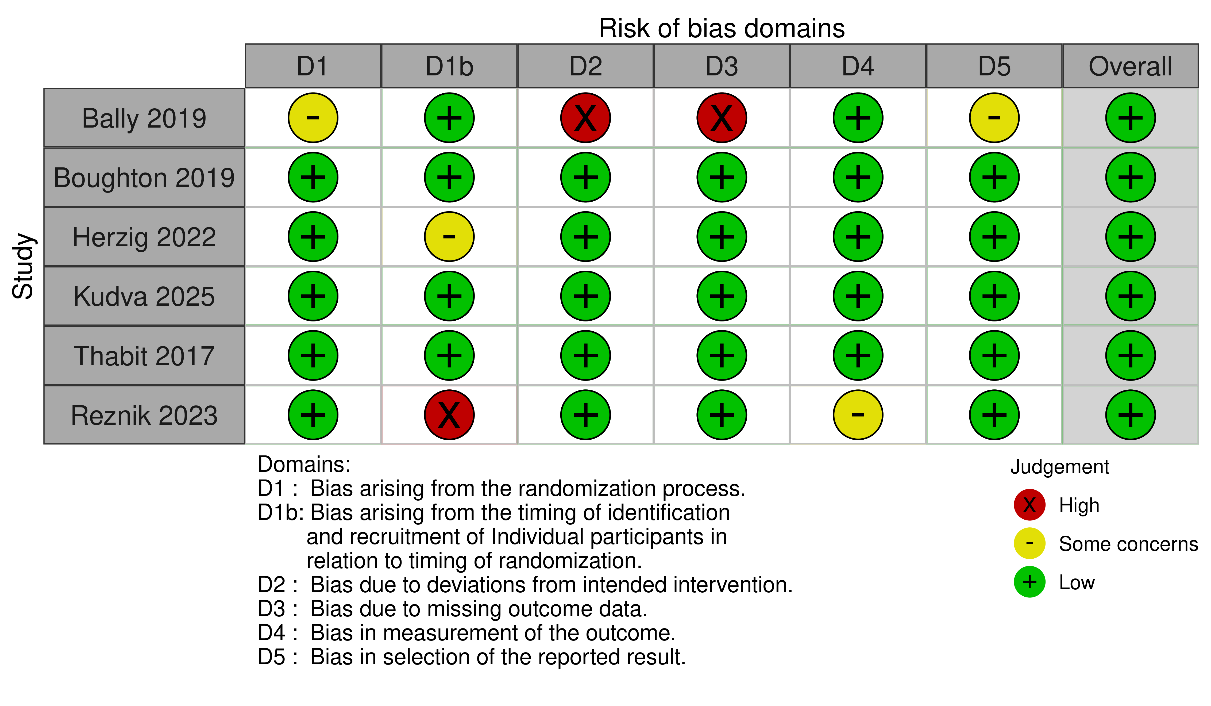


1. Crossover RCTs


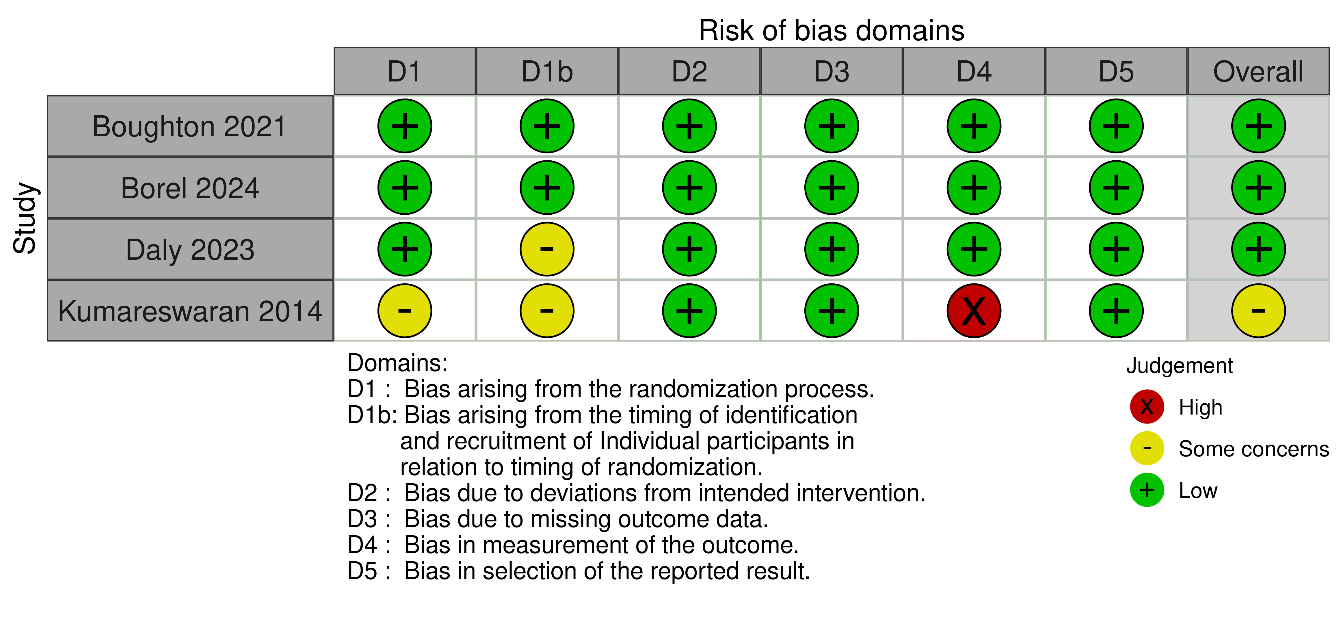


**Online supplementary material 2:** Forest Plots

**Figure S3.** Total Daily Insulin

1. Overall


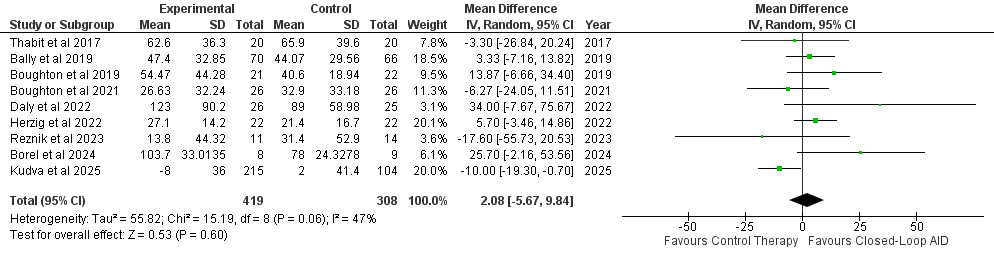


1. Short-term vs Long-term duration


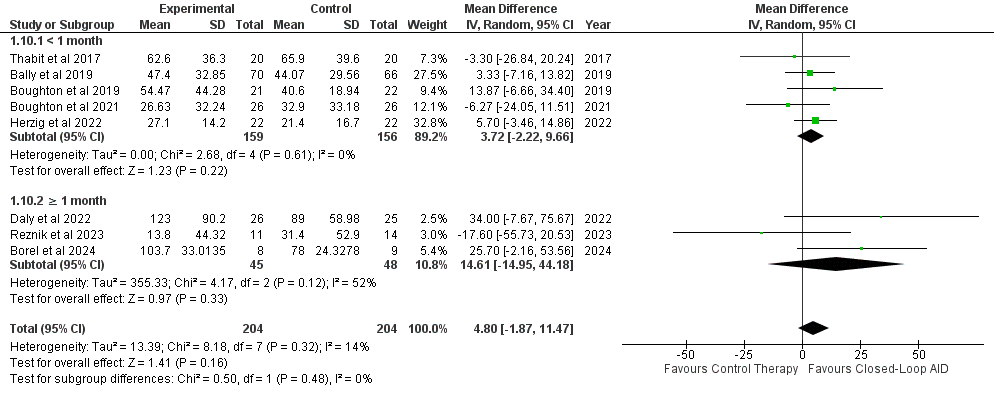


**Figure S4.** Serious Adverse Events

1. Overall


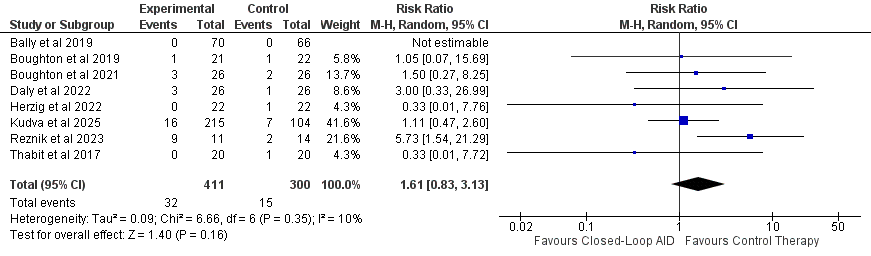


1. Short-term vs Long-term duration


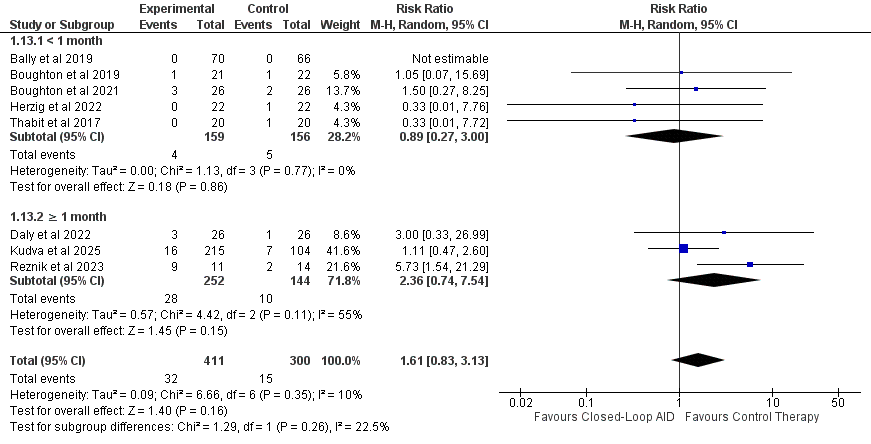


**Figure S5.** Severe Hypoglycemic event

1. Overall


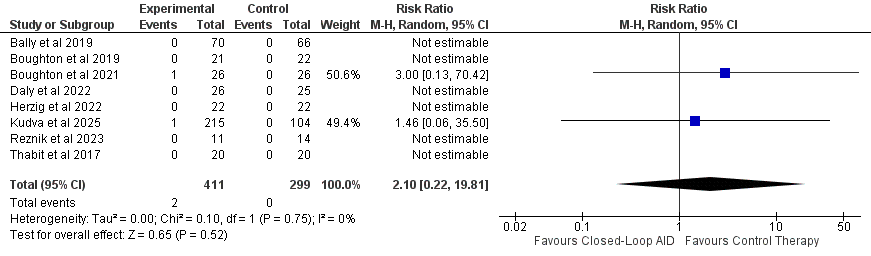


1. Short-term vs Long-term duration


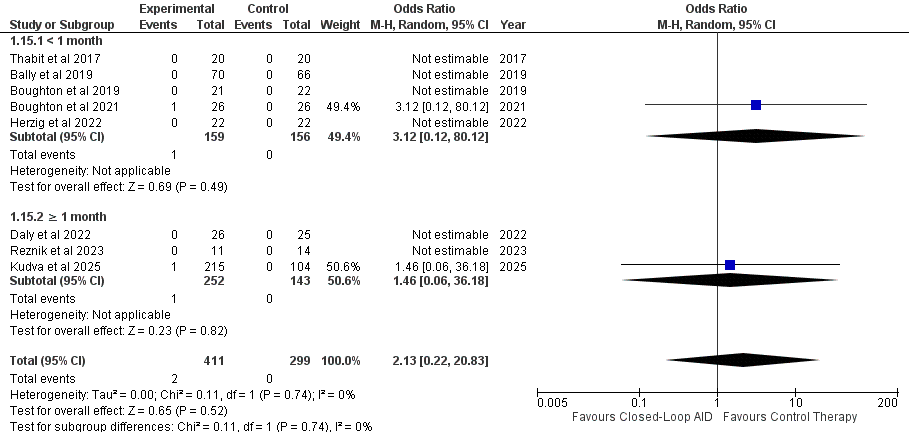


**Online supplementary material 3:** Forest plots subgrouped by patient population

**Figure S6: Time spent in normoglycemic range**

**
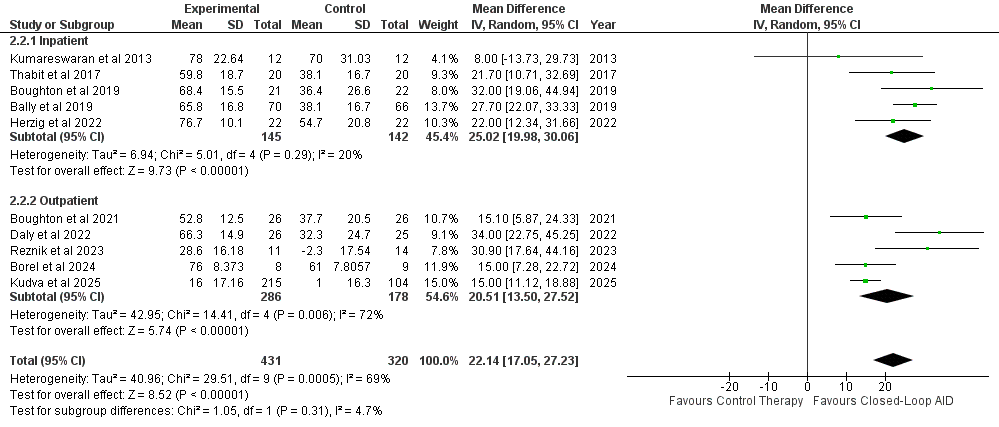
**

**Figure S7: Time spent in hypoglycemic range**

**
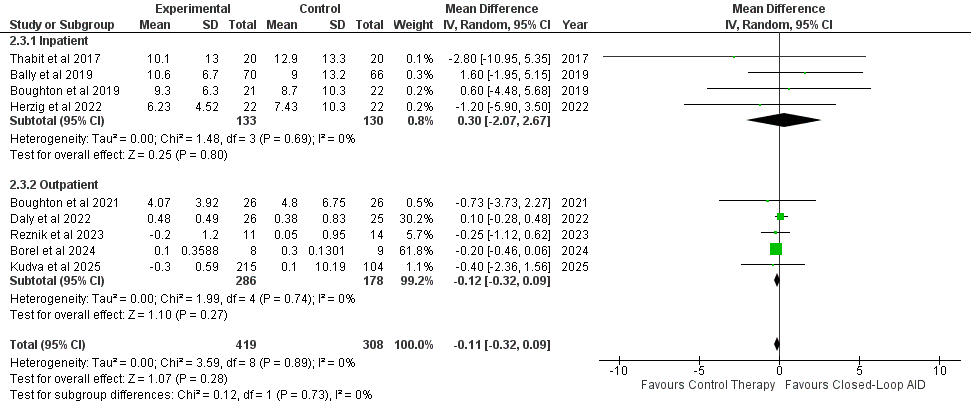
**

**Figure S8: Time spent in hyperglycemic range**

**
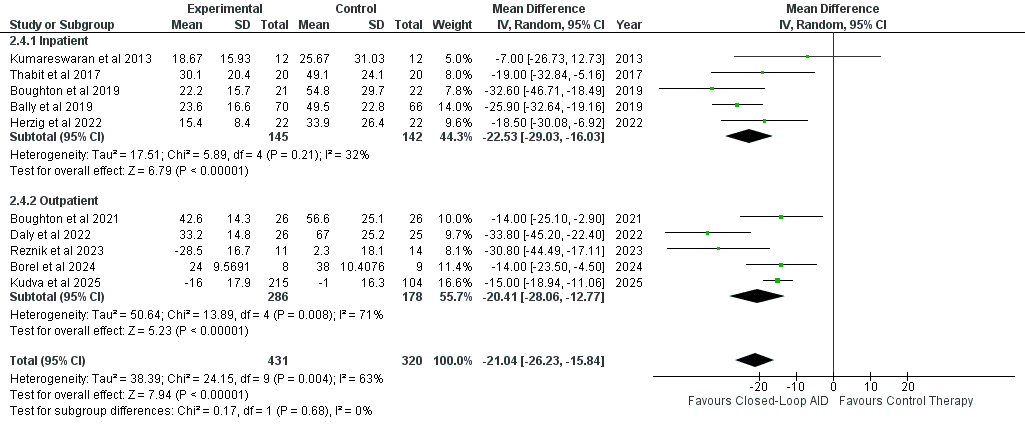
**

**Figure S9: Variability of glucose (SD)**

**
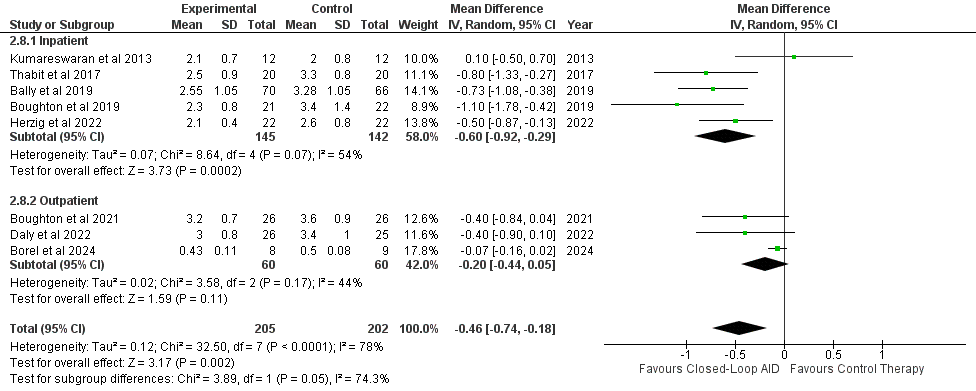
**

**Figure S10: Coefficient of variation (CV) of glucose**

**
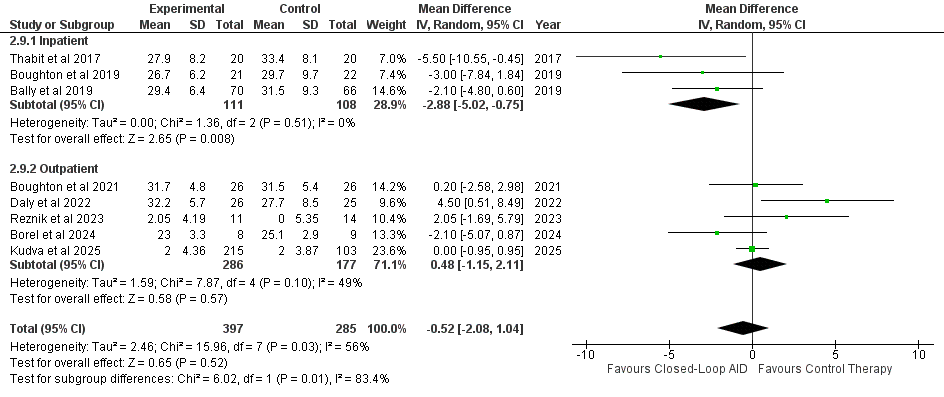
**

**Figure S11: Mean glucose levels**


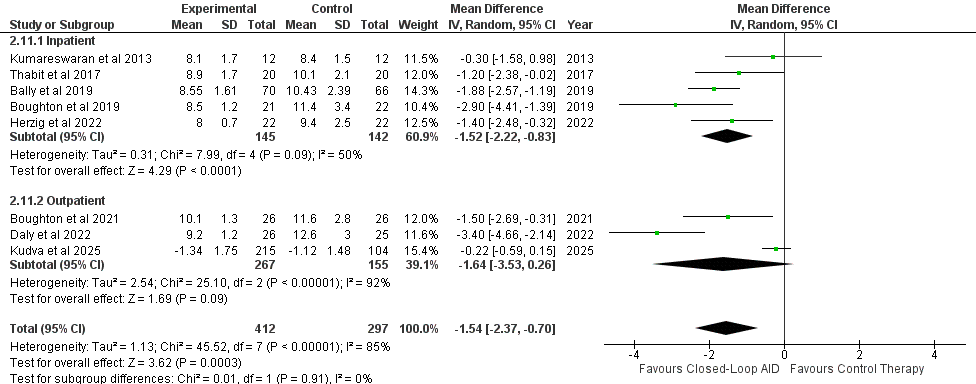


**Figure S12: Total adverse events**

**
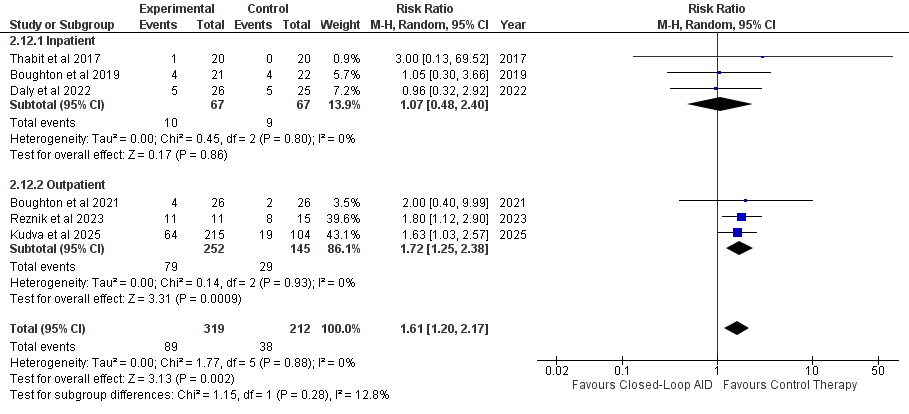
**

**Figure S13: Serious adverse events**

**
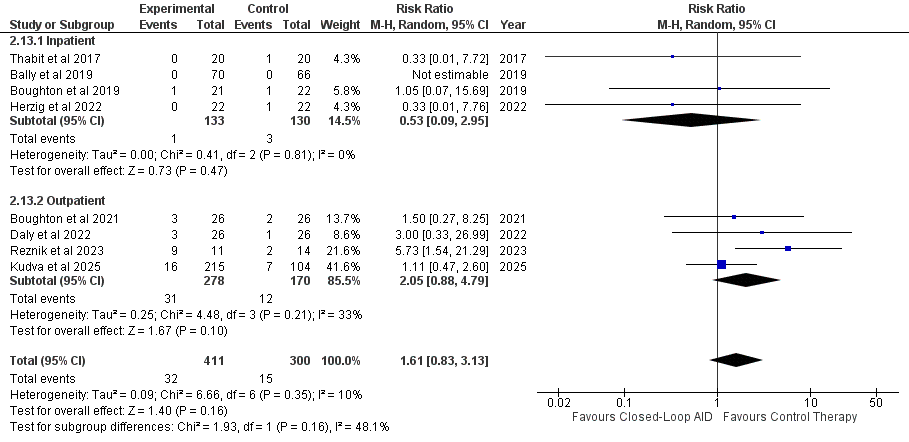
**

**Online supplementary material 4:** Sensitivity Analysis plots

**Figure S14.** Time Spent Near Normoglycemic Range after exclusion of Borel et al 2024 and Kudva et al 2025.


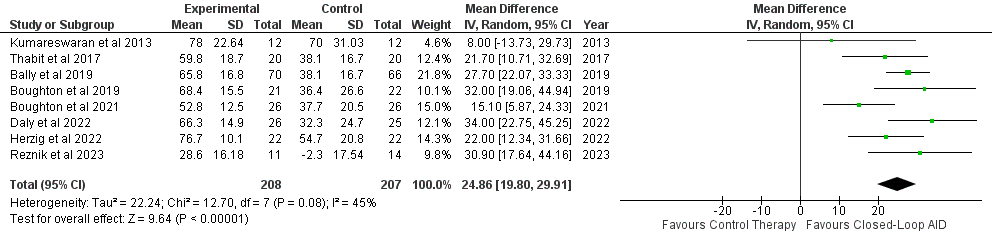


**Figure S15.** Time Spent Near Hyperglycemic Range after exclusion of Borel et al 2024 and Kudva et al 2025**.**


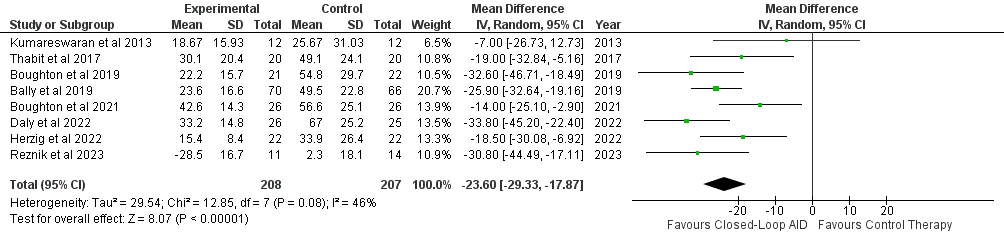


**Figure S16.** Variability of glucose (SD) after exclusion of Borel et al 2024.


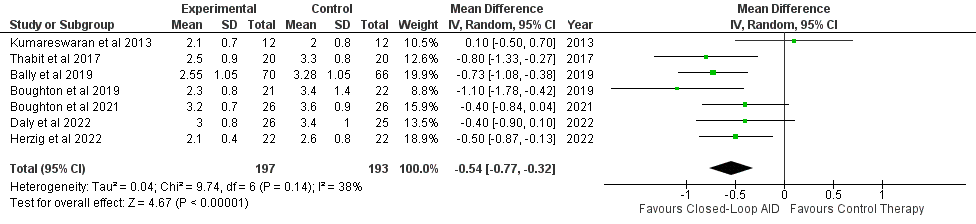


**Figure S17.** Coefficient or variation (CV) in glucose level after exclusion of Daly et al 2022.


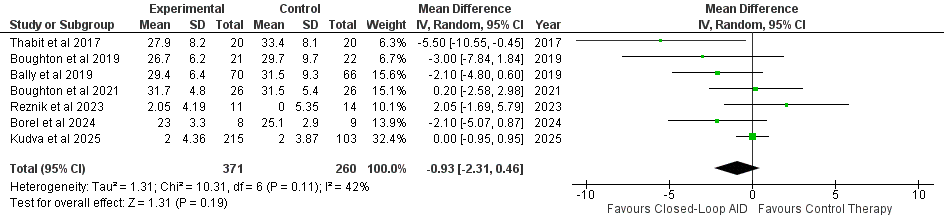


**Figure S18.** Mean glucose levels after exclusion of omitting Daly et al 2022 and Kudva et al 2025.


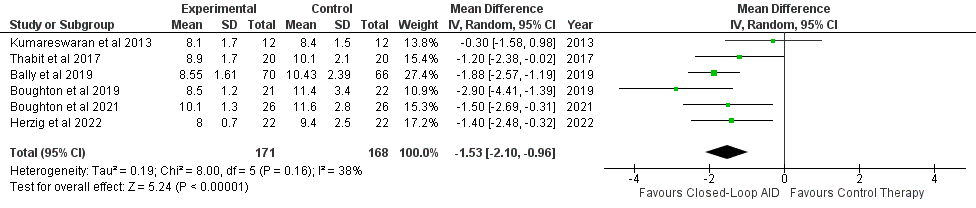


**Online supplementary material 5:** Egger’s publication bias plots

**Figure S19.** Publication bias measured by Egger’s test on the time spent in normoglycemic range


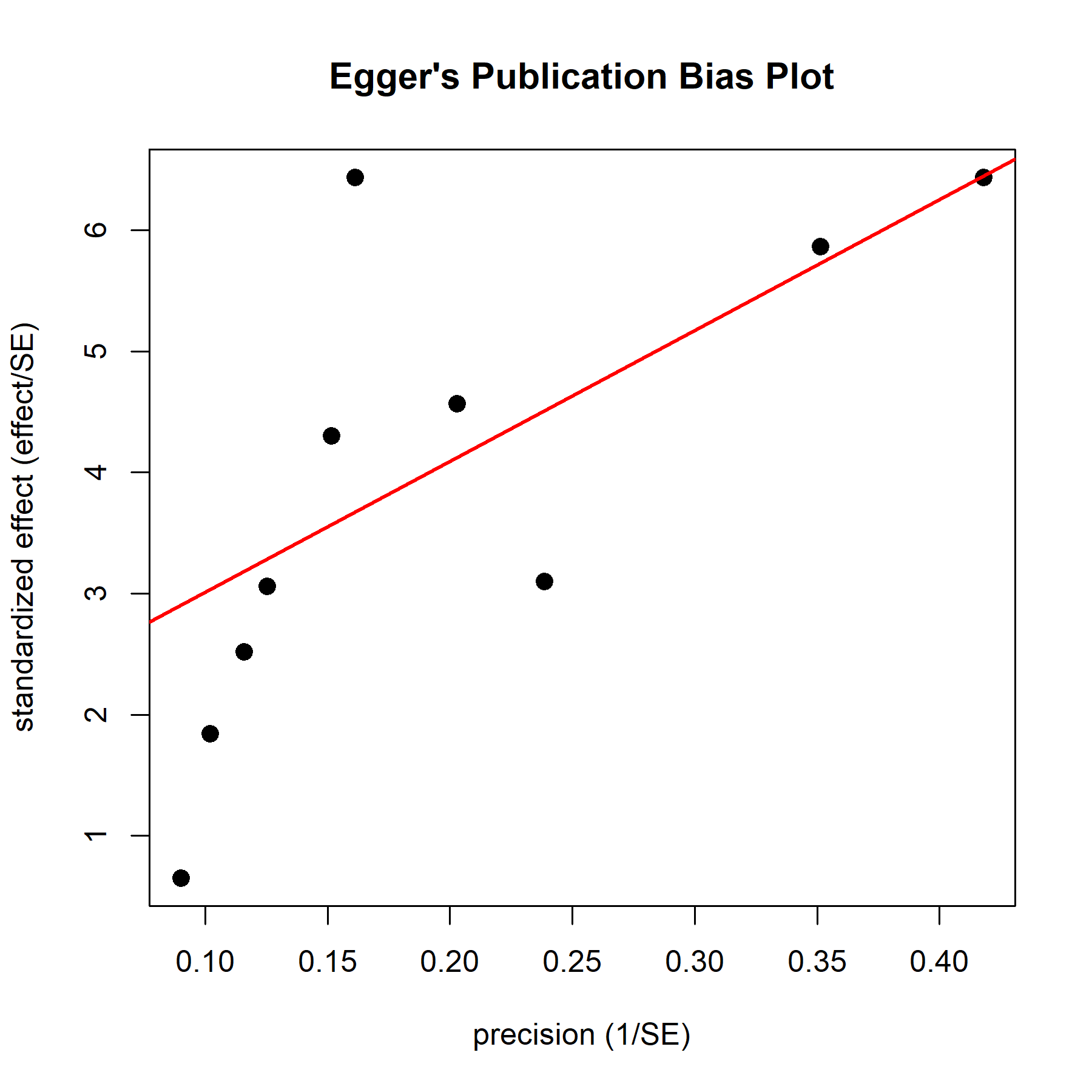


**Figure S20.** Publication bias measured by Egger’s test on the time spent in hyperglycemic range


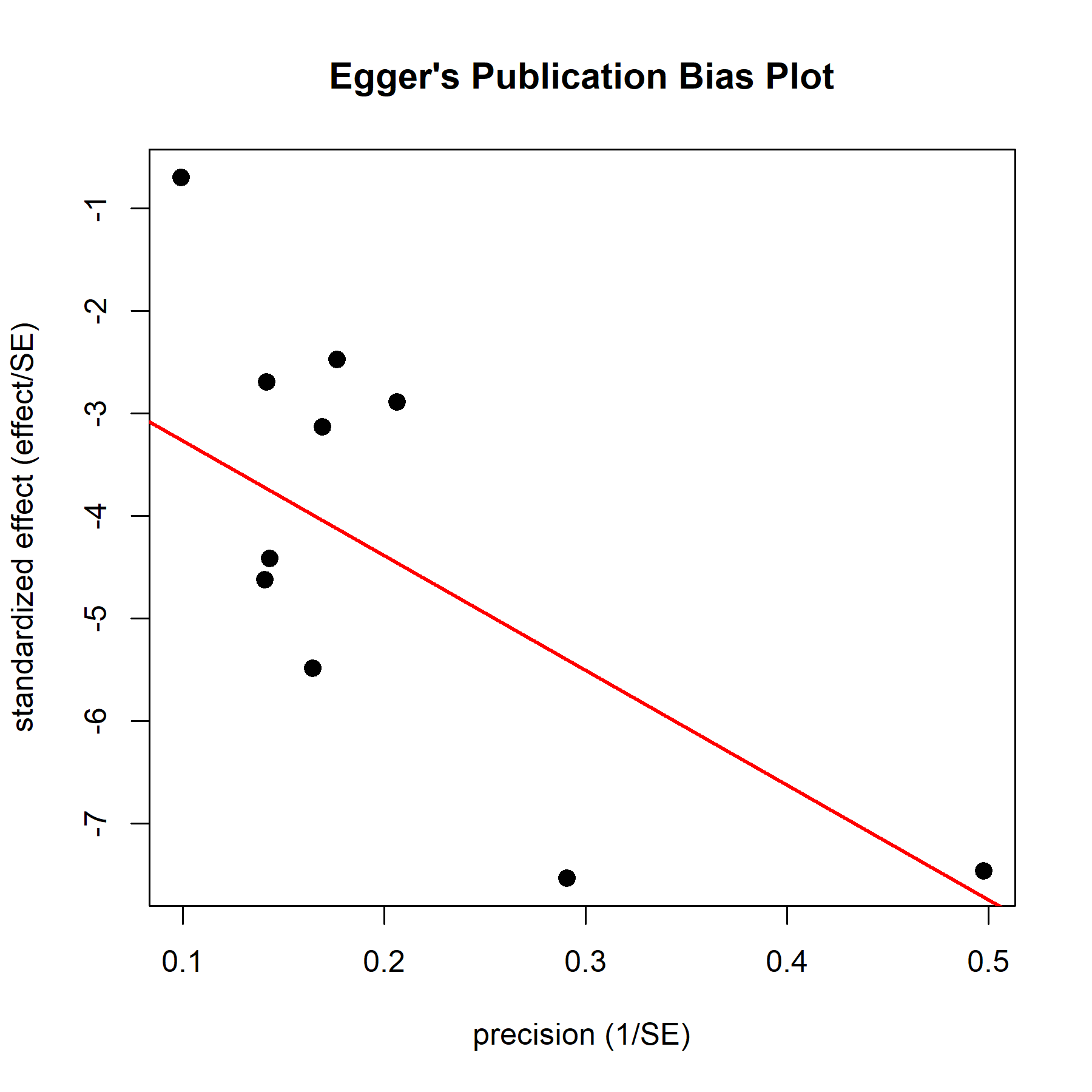

Supplement: Supplementary file 1 — Appendix S1: edm270178‐sup‐0001‐AppendixS1.docx. Table S1: Detailed search string for extracted studies form databases. Figure S1: PRISMA flowchart. Figure S2: Risk of bias assessment of the included RCTs. Figure S3: Total Daily Insulin. Figure S4: Serious Adverse Events. Figure S5: Severe Hypoglycemic event. Figure S6: Time spent in normoglycemic range. Figure S7: Time spent in hypoglycemic range. Figure S8: Time spent in hyperglycemic range. Figure S9: Variability of glucose (SD). Figure S10: Coefficient of variation (CV) of glucose. Figure S11: Mean glucose levels. Figure S12: Total adverse events. Figure S13: Serious adverse events. Figure S14: Time Spent Near Normoglycemic Range after exclusion of Borel et al. 2024 and Kudva et al. 2025. Figure S15: Time Spent Near Hyperglycemic Range after exclusion of Borel et al. 2024 and Kudva et al. 2025. Figure S16: Variability of glucose (SD) after exclusion of Borel et al. 2024. Figure S17: Coefficient or variation (CV) in glucose level after exclusion of Daly et al. 2022. Figure S18: Mean glucose levels after exclusion of omitting Daly et al. 2022 and Kudva et al. 2025. Figure S19: Publication bias measured by Egger's test on the time spent in normoglycemic range. Figure S20: Publication bias measured by Egger's test on the time spent in hyperglycemic range. [file EDM2-9-e70178-s001.docx]
